# Supplementary material for: Wearable Sensors as a Preoperative Assessment Tool: A Review
Source: Sensors (Basel). 2024 Jan 12;24(2):482. doi: 10.3390/s24020482 (PMC10820534; doi:10.3390/s24020482)
Supplement: Supplementary file 1 [file sensors-24-00482-s001.zip › Supplementary Table S1_upload.pdf]

Supplementary Table S1.

Title	Sample size	Average age	Gender split (% male)	Ethnicity	Health condition
Predicting Outcomes in Patients Undergoing Pancreatectomy Using Wearable Technology and Machine Learning: Prospective Cohort Study	48	63.2	40%	White: 95%<br>Non-white: 5%	Patients undergoing pancreatectomy
Predicting Post-Operative Complications with Wearables: A Case<br>Study with Patients Undergoing Pancreatic Surgery	61	64.4	69%	White/Caucasian: 92%<br>Black or African American: 0.5%<br>Unknown: 3%	Patients undergoing pancreatectomy
Objectively measured preoperative physical activity is associated with time to functional recovery after hepato-pancreato-biliary cancer surgery: a pilot study	31	66	58%	N/A	Hepato-pancreato-biliary cancer
Preoperative physical activity levels and postoperative pulmonary complications post-esophagectomy	37	61	78%	N/A	Esophageal cancer
Feasibility and patient's experiences of perioperative telemonitoring in major abdominal surgery: an observational pilot study	42	68	70%	N/A	Patients undergoing major abdominal surgery
Wearable Technology in the Perioperative Period: Predicting Risk of Postoperative Complications in Patients Undergoing Elective Colorectal Surgery	99	55	48.4%	White: 89%	Patients undergoing elective colorectal surgery
Fitbit Data to Assess Functional Capacity in Patients Before Elective Surgery: Pilot Prospective Observational Study	31	76	29%	N/A	Patients under consideration for major non-cardiac surgeries
Wearable Health Technology for Preoperative Risk Assessment in	31	76	29%	N/A	Patients under consideration for major non-cardiac surgeries

Supplementary Table 1.

Elderly Patients: The WELCOME Study					
Wearable technology and the association of perioperative activity level with 30-day readmission among patients undergoing major colorectal surgery	94	54	48%	White: 89%	Patients undergoing major elective colorectal surgery
Wireless Monitoring Program of Patient-Centered Outcomes and Recovery Before and After Major Abdominal Cancer Surgery	20	55	25%	White: 65%	Patients scheduled to undergo curative resection for hepatobiliary and GI cancers
Predicting post-discharge cancer surgery complications via telemonitoring of patient-reported outcomes and patient-generated health data	52	N/A	N/A	N/A	Patients with Gastrointestinal or Lung cancer
The association between low pre-operative step count and adverse post-operative outcomes in older patients undergoing colorectal cancer surgery	85	76	49%	White: 92%<br>Maori: 3.5%<br>Other: 4.7%	Patients undergoing surgery for major colorectal cancer surgery
How Many Steps Per Day are Necessary to Prevent Postoperative Complications Following Hepato-Pancreato-Biliary Surgeries for Malignancy?	78	71	68%	N/A	Patients scheduled to undergo open abdominal surgeries for HPB malignancies
Preoperative Physical Activity Predicts Surgical Outcomes Following Lung Cancer Resection	78	70	45%	N/A	Patients undergoing anatomical Lung resection
Modeling Biobehavioral Rhythms with Passive Sensing in the Wild: A Case Study to Predict Readmission Risk after Pancreatic Surgery	53	65	47%	White: 94%	Patients undergoing surgery for pancreatic cancer or benign conditions (e.g. pancreatic cysts).

Supplementary Table 1.

Value of the average basal daily walked distance measured using a pedometer to predict maximum oxygen consumption per minute in patients undergoing lung resection	38	63	79%	N/A	Patients referred to major lung resection for lung cancer
Prediction of Physiological Response over Varying Forecast Lengths with a Wearable Health Monitoring Platform	N/A	N/A	N/A	N/A	N/A
Learning Individualized Cardiovascular Responses from Large-scale Wearable Sensors Data	80,137	31	18%	N/A	N/A
Self-supervised transfer learning of physiological representations from free-living wearable data	2,100	N/A	N/A	N/A	N/A
Wearable sensors enable personalized predictions of clinical laboratory measurements	54	57	44%	European: 74%<br>Asian: 15%<br>African American:7%<br>Hispanic: 4%	N/A
Turning silver into Gold:Domain adaptation with noise labels for wearable cardio-respiratory fitness prediction	12,425	N/A	N/A	N/A	N/A
Cardiorespiratory fitness estimation in free-living using wearable sensors	46	25	84%	N/A	N/A
Cardiorespiratory fitness estimation using wearable sensors: Laboratory andfree-living analysis of context-specific submaximal heart rates	51	25	88%	N/A	N/A
Prediction of oxygen uptake dynamics by machine learning	16	27	100%	N/A	N/A

Supplementary Table 1.

analysis of wearable sensors during activities of daily living					
Longitudinal cardio-respiratory fitness prediction through wearables in free-living environments	11,059	N/A	48%	N/A	N/A
The association of pre-operative home accelerometry with cardiopulmonary exercise variables	48	71	88%	N/A	Patients attending the pre-operative cardiopulmonary exercise clinic
Can wearable technology be used to approximate cardiopulmonary exercise testing metrics?	49	65	65%	N/A	Patients scheduled for major elective intra-abdominal surgery

Supplementary Table S1. This table provides a breakdown of the samples used in each of the key research papers included in this review for analysis. Within each column, the sample size is reported alongside participant demographics including: Average age, gender split, ethnicity and health outcomes.
