# Supplementary material for: Wearable Sensors as a Preoperative Assessment Tool: A Review
Source: Sensors (Basel). 2024 Jan 12;24(2):482. doi: 10.3390/s24020482 (PMC10820534; doi:10.3390/s24020482)
Supplement: Supplementary file 1 [file sensors-24-00482-s001.zip › Supplementary Table S2_upload.pdf]

Supplementary Table S2.

Title	Authors	Year	Outcome	Sensor & Sensor Modality	Pre-processing Techniques	Features Extracted	Analysis Models
Predicting Outcomes in Patients Undergoing Pancreatectomy Using Wearable Technology and Machine Learning: Prospective Cohort Study	Cos et al.	2021	Complications post pancreatectomy	Fitbit Inspire HR:<br>- PPG<br>- Accelerometer	Detrended Fluctuation Analysis (DFA)<br>2 level imputation method for missing data	Daily Step Count<br>Heart rate<br>Sleep time-series	RF<br>GBT<br>KNN<br>SVM<br>Logistic Regression
Predicting Post-Operative Complications with Wearables: A Case Study with Patients Undergoing Pancreatic Surgery	Zhang et al.	2022	Complications post pancreatectomy	Fitbit Inspire HR:<br>- PPG<br>- Accelerometer	Daily feature extraction threshold > 8hours.<br>2 level imputation method for missing data (Figure 7):<br>Short term imputation (<10mins)<br>Robust singular spectrum analysis to impute missing daily features	Daily Feature extraction:<br>Step<br>Sleep<br>HR time series<br><br>High level feature extraction:<br>Singular spectrum analysis	RF<br>XGBoost<br>KNN<br>SVM<br>Lasso Regressions<br>Ridge Regression
Objectively measured preoperative physical activity is associated with time to functional recovery after hepato-pancreato-biliary cancer surgery: a pilot study	Mylius et al.	2021	Time to functional recovery post hepato-pancreato-biliary cancer surgery	Actigraph wGT3X- BT+:<br>- Accelerometer	Extraction Threshold of 6 days+ of data	Time spent in MVPA: daily median of total accumulated minutes, daily median minutes accumulate in >10 minute bouts	Univariate and multivariate robust regression
Preoperative physical activity levels and postoperative pulmonary complications post-esophagectomy	Feeney et al.	2011	Postoperative pulmonary complications post-esophagectomy	RT3 Accelerometer:<br>- Accelerometer	N/A	Physical activity intensities: inactive, light, moderate and vigorous	Independent t-test
Feasibility and patient's experiences of perioperative telemonitoring in major abdominal surgery: an observational pilot study	Haveman et al.	2022	Compliance and satisfaction with wearable sensor	Everion Biosensor:<br>- PPG<br>- Accelerometer	N/A	N/A	N/A
Wearable Technology in the Perioperative Period: Predicting Risk of Postoperative Complications in Patients Undergoing Elective Colorectal Surgery	Hedrick et al.	2020	Postoperative complications	Fitbit charge 2:<br>- PPG<br>- Accelerometer	N/A	Daily steps and stratification into two groups (active:>5000 dailys steps, inactive<5000)<br>Daily HR	Chi-squared<br>Wilcoxon rank-sum<br>Kruskal-Wallis<br>Multivariable regression models

Supplementary Table 2.

Fitbit Data to Assess Functional Capacity in Patients Before Elective Surgery: Pilot Prospective Observational Study	Angelucci et al.	2023	6MWT	Fitbit Inspire 2:<br>- PPG<br>- Accelerometer	Missing PPG data imputed with HR data from hospital visit.	HR<br>RHR (24hr average)<br>HR Zones during exercise<br>Daily steps<br>Distance walked<br>Physical activity intensities<br>HROS<br>NET-F	Pearson correlation<br>Wilcoxon Rank Sum Test
Wearable Health Technology for Preoperative Risk Assessment in Elderly Patients: The WELCOME Study	Greco et al.	2023	6MWT<br>Preoperative scales	Fitbit Inspire 2:<br>- PPG<br>- Accelerometer	N/A	Average daily steps<br>VO2max (as processed by the Fitbit device)<br>HR data<br>activity intensity<br>energy expenditure<br>calories.	Correlation analyses
Wearable technology and the association of perioperative activity level with 30-day readmission among patients undergoing major colorectal surgery	Kane et al.	2022	30-day readmission	Fitbit Charge 2:<br>- PPG<br>- Accelerometer	N/A	Daily Step count<br>Average HR	Chi-squared/Fishers exact test (categorical)<br>Wilcoxon Rank Sum/Kruskal Wallis (continuous)
Wireless Monitoring Program of Patient-Centered Outcomes and Recovery Before and After Major Abdominal Cancer Surgery	Sun et al.	2017	Adherence with WS<br>Satisfaction with monitoring	Garmin Vivofit 2:<br>- Accelerometer	N/A	Patients Daily Steps	Correlation
Predicting post-discharge cancer surgery complications via telemonitoring of patient-reported outcomes and patient-generated health data	Rossi et al.	2021	Complications up to 30 days post discharge	Garmin Vivofit:<br>- Accelerometer	N/A	Daily steps: maximum, minimum, SD, medium, slope and intercept of linear interpolation, differences compared to baseline	Logistic Regression
The association between low pre-operative step count and adverse post-operative outcomes in older patients undergoing colorectal cancer surgery	Richards et al.	2020	Length of hospital stay<br>Rate of postoperative complications<br>Mortality	Garmin Vivofit 3:<br>- Accelerometer	N/A	Patients stratified into two groups from step count:<br><2500:low, >2500:normal	Univariate variables:<br>Chi-sqaured and Kruskal-Wallis<br>Negative Binomial Regressions<br>Multivariable Logistic Regression

Supplementary Table 2.

How Many Steps Per Day are Necessary to Prevent Postoperative Complications Following Hepato-Pancreato-Biliary Surgeries for Malignancy?	Nakajima et al.	2020	Rate of major complications with Clavien-Dindo complications<br>Rate of infectious complications<br>Length of hospital stay	Kenx LifecoderGZ:<br>- Accelerometer	N/A	Patients stratified into two groups from step count: <5000:poor, ≥ 5000:good	Chi-sqaured and Fisher's exact test and Mann-Whitney *U* test<br>Spearman's rank correlation coefficient<br>Multivariate logistic regression
Preoperative Physical Activity Predicts Surgical Outcomes Following Lung Cancer Resection	Billé et al.	2021	Respiratory and cardiac complications<br>30-day readmission rate	3D Trisport:<br>- Accelerometer	N/A	Patients split into 4 groups based on daily step count based on median and 25% quartiles (not pre-defined quarters).	Chi-squared or 2-tailed T-tests
Modeling Biobehavioral Rhythms with Passive Sensing in the Wild: A Case Study to Predict Readmission Risk after Pancreatic Surgery	Doryab at el.	2019	Re-admission within 90-days of discharge.	Fitbit Charge 2:<br>- PPG<br>- Accelerometer		From step count:<br>Number, length and number of steps in active bouts<br>Number and length of sedentary bouts<br>From HR:<br>The minimum, maximum and mean of positive, negative and absolute change in HR<br><br>Detection of rhythmicity by building individual's cosinors using data from each patient before build population level cosinors from readmitted vs non-readmitted patients.<br>Visual features were also extracted from autocorrelation and periodograms	RF<br>Logistic Regression<br>SVM<br>Bayesian Network<br>Boosted Logistic Regression.
Value of the average basal daily walked distance measured using a pedometer to predict maximum oxygen consumption per minute in patients undergoing lung resection	Novoa et al.	2010	VO_{2Max}	OMROM walking style pedometer PRO:<br>- Accelerometer	N/A	Daily steps<br>Daily aerobic steps (aerobic steps are calculated after 10 mins of walking at >60steps per min)<br>Daily time spent in aerobic activity (minutes)<br>Daily distance measured (km)	Linear Regression Models<br>Plotting of correlation index between models using Bland and Altman method.

Supplementary Table 2.

Prediction of Physiological Response over Varying Forecast Lengths with a Wearable Health Monitoring Platform	Mohammadzadeh et al.	2018	Breathing rate	Bioharness Zephyr:<br>- ECG<br>- Accelerometer<br>Empatica E4:<br>- PPG<br>- Accelerometer	N/A	Respiratory rate<br>HR<br>HRV	SVM
Learning Individualized Cardiovascular Responses from Large-scale Wearable Sensors Data	Hallgrimson et al.	2018	Cardiovascular response<br>Age<br>BMI*	Achievement reward platform (Fitbit or Apple watch):<br>- Accelerometer<br>- PPG	Minute level from step count and heart rate was scaled to measurements between (0,1).<br>Missing data was imputed as mean HR of activity at waking hours.	HR<br>Step count<br>Two sleep stages: Asleep or restless asleep	HR autoencoder that is trained on physical activity and sleep stages. The signature encoder learns the participants 'signature' from the HR responses to activity and the decoder employs the learned signature predict HR from PA.<br><br>XGBoost models were used to compare performance against the encoder.
Self-supervised transfer learning of physiological representations from free-living wearable data	Spathis et al.	2021	HR Response<br>VO_2 Max<br>BMI<br>Resting HR	Actiheart Chest ECG wearable monitor (2-lead)<br>- ECG<br>Wrist device triaxial accelerometer<br>- Accelerometer	Extraction threshold of >72 hours for inclusion.<br>Magnitude of acceleration from accelerometer data was calculated through Euclidean Norm Minus One and high passed filtered vector magnitude. Both accelerometer and HR data was filtered to a time resolution of one sample per 15 seconds.<br>Temporal features were encoded into timestamps using cyclical temporal features.	HR<br>Acceleration	Proposal of a multimodal self-supervised model for feature extraction from wearable data. The 'Step2Heart' model receives high-dimensional activity inputs to predict HR response. It stacks CNN and RNN layers where the CNN learns spacial features and the RNN learns temporal features of the data.<br>This is compared against several models:<br>Convolutional autoencoder<br>XGBoost

Supplementary Table 2.

Wearable sensors enable personalized predictions of clinical laboratory measurements	Dunn et al.	2021	Clinical laboratory measurements including:<br>Hematocrit<br>Hemoglobin<br>Red blood cell counts<br>Absolute monocyte counts<br>HbA1c (average blood glucose)	Intel Basis Smartwatch:<br>- PPG<br>- Accelerometer<br>- Skin Temperature<br>EDA	Implementation of same method for pre-processing clinical records: removal of outliers defined as values >3 S.D. from the mean for that laboratory.	153 features from the continuous wearable sensor data.	RF<br>Two-sided Wilcoxon signed Rank test.
Turning silver into Gold:Domain adaptation with noise labels for wearable cardio-respiratory fitness prediction	Wu et al.	2022	VO_2Max	Actiheart Chest ECG wearable monitor (2-lead)<br>- ECG<br>Wrist device triaxial accelerometer<br>- Accelerometer	Non-wear periods were removed through pre-processing algorithm that identified periods of non-physical heart rate and no movement.<br>Movement intensities were converted into standard metabolic equivalent units (METs).<br>Signals were down sampled to a frequency of 15 minutes.	HR<br>Acceleration	Proposal of UDAMA : Unsupervised Domain Adaptation and Multi-Discriminator Adversarial Training that uses the noisy data that is labelled 'silver standard' to improve the modelling of gold standard data.
Cardiorespiratory fitness estimation in free-living using wearable sensors	Altini et al.	2016	VO_2Max	Holst ECG Necklace:<br>- ECG<br>- Accelerometer	Accelerometer data was band-passed between 0.1 and 10Hz to isolate dynamic components. HR was extracted from RR intervals and averaged over 15s.	From accelerometer:<br>Mean of absolute signal<br>Interquartile range<br>Median, variance<br>Low frequency band signal power.<br>HR	Hierarchical Bayesian models for cardio-respiratory fitness estimation.
Cardiorespiratory fitness estimation using wearable sensors: Laboratory andfree-living analysis of context-specific submaximal heart rates	Altini et al.	2016	VO_2Max	Holst ECG Necklace:<br>- ECG<br>- Accelerometer	For the detection of activities recognised as walking, the accelerometer signal was segmented to 5s and filtered by two separate filters. HR was averaged over 15s.	From accelerometer:<br>Mean of absolute signal<br>Interquartile range<br>Median, variance<br>Low frequency band signal power.<br>HR	Multiple Linear Regressions models using LOPO cross validation.
Prediction of oxygen uptake dynamics by machine learning	Beltrame et al.		VO_2	Hexoskin smartshirt:<br>- ECG<br>- Accelerometer	The HR difference variable was calculated by finding the difference	HR<br>Difference in HR<br>Total hip accereleration	RF

Supplementary Table 2.

analysis of wearable sensors during activities of daily living				- Respiration Band	between the current HR value and the previous value. Features were low-pass filtered 0.01Hz.	Minute ventilation<br>Breathing frequency<br>Walking cadence	
Longitudinal cardio-respiratory fitness prediction through wearables in free-living environments	Spathis et al.	2022	VO_2Max	Actiheart Chest ECG wearable monitor (2-lead)<br>- ECG<br>Wrist device triaxial accelerometer<br>- Accelerometer	Non-wear periods were removed through pre-processing algorithm that identified periods of non-physical heart rate and no movement.<br>Movement intensities were converted into standard metabolic equivalent units (METs).<br>Signals were down sampled to a frequency of 15 minutes.<br>Temporal factors were encoded into sensor timestamps.	Statistical features were extracted from signals and every participant week was represented as a row in the feature vector.<br>Features included:<br>Acceleration<br>HR<br>HRV<br>Acceleration derived Euclidean Norm Minus One<br>Acceleration Derived METs.	Deep Neural Network, a network of 2 densely connected feed-forward layers with 128 units.
The association of pre-operative home accelerometry with cardiopulmonary exercise variables	Cui et al.	2017	VO_2Max<br>Anaerobic Threshold (AT)	AX3 Axivity:<br>- Accelerometer	Employed a fast Fourier transformation to integrate the frequency between 1Hz and 10Hz of the power spectrum.<br>The extraction threshold was set at above 23hrs per day. Each 10s period was categorised as active, stationary or lying.	Activity score<br>Acceleration (mean, S.D., lateral axis mean, vertical axis mean, frequency).	Multiple linear regression analysis.
Can wearable technology be used to approximate cardiopulmonary exercise testing metrics?	Jones et al.	2021	VO_{2peak}<br>Ventilatory equivalent for CO_2<br>AT<br>Peak work	Garmin Vivosmart HR:<br>- Accelerometer<br>- PPG	Features were averaged across the 7-day wear period. Total METs was calculated by summing METs from across the week.	HR<br>Average HR, Maximum HR<br>Total steps<br>Floors climbed<br>Number of intense minutes of exercise<br>Total calories<br>Total distance travelled	Linear Regression<br>Correlation between fitted and observed values.

Supplementary Table S2. This table provides a comprehensive breakdown of the most relevant papers included in this review. The columns present the data extracted from each paper including the Outcome variable, sensor modality, pre-processing methods, features extracted and the model of analysis.

Supplementary Table 2.
